# Coral recovery in the central Maldives archipelago since the last major mass-bleaching, in 1998

**DOI:** 10.1038/srep34720

**Published:** 2016-10-03

**Authors:** C. Pisapia, D. Burn, R. Yoosuf, A. Najeeb, K. D. Anderson, M. S. Pratchett

**Affiliations:** 1 ARC Centre of Excellence for Coral Reef Studies, James Cook University, Townsville QLD 4811, Australia; 2 Gili Lankanfushi Island, North Male Atoll, Republic of Maldives; 3 Marine Research Centre, Ministry of Fisheries Agriculture and Marine Resources, Moonlight Hingun, 20025, Male’ Republic of Maldives

## Abstract

Increasing frequency and severity of disturbances is causing global degradation of coral reef ecosystems. This study examined temporal changes in live coral cover and coral composition in the central Maldives from 1997 to 2016, encompassing two bleaching events, a tsunami, and an outbreak of *Acanthaster planci*. We also examined the contemporary size structure for five dominant coral taxa (tabular *Acropora*, *Acropora muricata*, *Acropora humilis*, *Pocillopora spp*, and massive *Porites*). Total coral cover increased throughout the study period, with marked increases following the 1998 mass-bleaching. The relative abundance of key genera has changed through time, where *Acropora* and *Pocillopora* (which are highly susceptible to bleaching) were under-represented following 1998 mass-bleaching but increased until outbreaks of *A. planci* in 2015. The contemporary size-structure for all coral taxa was dominated by larger colonies with peaked distributions suggesting that recent disturbances had a disproportionate impact on smaller colonies, or that recruitment is currently limited. This may suggest that coral resilience has been compromised by recent disturbances, and further bleaching (expected in 2016) could lead to highly protracted recovery times. We showed that Maldivian reefs recovered following the 1998 mass-bleaching event, but it took up to a decade, and ongoing disturbances may be eroding reef resilience.

Disturbances play an important role in the development, structure and function of natural communities and are a necessary part of ecosystem dynamics^1^,^2^. Intermediate levels of disturbance make an important contribution to increasing biodiversity^3^. However, increasing anthropogenic stresses (e.g., agricultural land use, overfishing, and pollution) are compounding upon natural disturbances, and inevitably leading to degradation of both terrestrial and aquatic ecosystems^2^,^4^. Natural communities constantly experience some level of disturbance, and are always recovering from past disturbances^1^. Some species recover rapidly in the aftermath of disturbances and make substantial contributions to overall habitat structure, such ground cover or benthic cover^5^, while other longer-lived and slow growing species, may take centuries to regain pre-disturbance levels of abundance^6^.

The recovery and resilience of natural communities to major acute disturbances (e.g. fire, cyclones, droughts, bleaching or predation) depends upon the spatial and temporal scales of the disturbance, the disturbance history, contemporary community structure, as well as background rates of mortality^7^,^8^,^9^,^10^,^11^. Importantly, rates of recovery are typically measured based on the time it takes for either overall abundance of key groups of organisms (e.g., total coral cover) or the specific abundance of individual taxa to reach levels apparent immediately prior to the disturbance^5^,^12^, which can be very fast or slow depending upon relative abundance of fast- and slow-growing species^13^. Fast-growing tabular and branching corals, for example, are the primary habitat-forming species^14^ and are usually very common because they are able to rapidly re-colonise reef habitats following a disturbance^14^,^15^. However, these corals are also the most susceptible to acute disturbances such as coral bleaching^16^, outbreaks of *Acanthaster planci*^17^, and cyclones^18^. Other growth forms such as massive and columnar, even though they have slower growth and lower rates of population turnover, they tend to be more resistant to acute disturbances^19^.

It is also important to realize that recovery from periodic and unpredictable acute disturbances operates against a background of chronic and more persistent disturbances, which can exert significant influence on demographics and biological interactions^20^, thereby affecting susceptibility to, and recovery from, acute disturbances^21^. There are no periods free of disturbances on coral reefs, as is the case for most ecosystems^1^,^11^,^22^,^23^,^24^. It is becoming increasingly clear that corals are routinely subject to significant rates of injuries and indeed, whole colony mortality^11^,^24^, and these can negatively affect the recovery capacity of populations between major acute disturbances^22^.

Good appreciation of the cumulative impacts of multiple and often diverse disturbances on coral reef organisms^25^, as well as the capacity to effectively project consequences of future disturbances^26^ is fundamentally dependent upon access to long-term data. While there are some locations that benefit from established long-term monitoring programs (e.g., Australia’s Great Barrier Reef:)^27^, information on long-term changes in the abundance and structure of coral assemblages is increasingly being generated from analyses of meta-data^25^,^28^. While there is often strong recovery in the aftermath of major disturbances^25^,^29^,^30^,^31^ most long-term studies reveal sustained declines in live coral cover^27^,^28^. Moreover, there have been pronounced shifts in the structure of coral assemblages^17^,^25^,^32^.

In the aftermath of major disturbances, total coral cover can increase very rapidly, but this is often conditional upon rapid growth of remnant tabular and/ or branching corals^5^. Recovery of population structure (e.g. the size structure distribution of coral populations) and community structure (e.g., the proportional representation of slow growing corals) will inevitably, take much longer. Since demographic processes such as survival, growth and fecundity are strongly size-dependent in corals^33^, the size structure of coral populations is an important driver of their dynamics^34^,^35^. Given inherent population dynamics, coral populations typically comprise many small individuals and relatively few large colonies^34^. In highly perturbed environments (e.g., due to high levels of chronic disturbances or in the immediate aftermath of major acute disturbances) the size-structure of coral population is likely to be even more truncated than usual^13^,^35^,^36^, with obvious consequences for reproduction, recruitment and population growth.

The purpose of this study was to explore trajectories in coral cover and composition within the central Maldives archipelago, especially since the 1998 mass-bleaching event. Maldivian coral reefs are highly vulnerable to climate change and have been increasingly exposed to anthropogenic disturbances over the last two decades^37^. We examined temporal changes in live coral cover and coral composition (from 1997 to 2016) and also investigated the contemporary size structure of six dominant groups of corals. Importantly, we tested whether spatial variation in the contemporary structure of coral assemblages was related to differences in abiotic factors such as depth and reef typology (e.g., oceanic versus lagoonal reefs) to test whether such factors confer increased resilience on coral reef habitats^38^. Given widespread degradation of coral reef ecosystems there is increasing impetus for identifying specific reef types or environmental settings that confer increased resilience^38^, prioritizing conservation of such locations to mediate longer-term effects of changing disturbances regimes and importantly assessing the fate of coral assemblages given the recurrence of mass bleaching in 2016^39^.

## Results

### Long-term changes in coral cover

**Figure 1:**
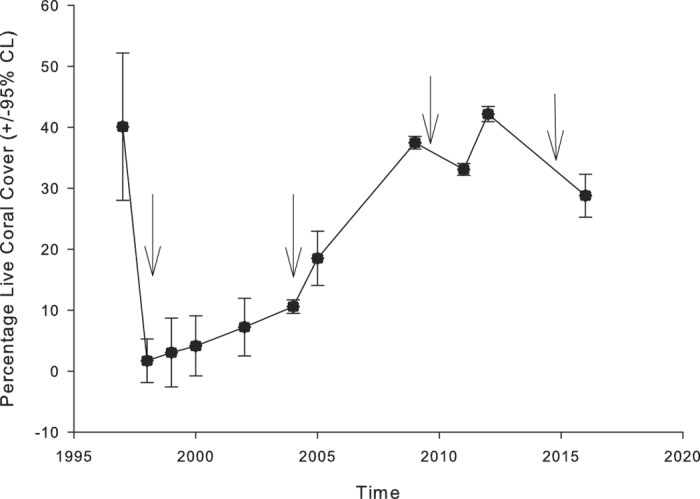
Variation in mean (±95% CL) coral cover at 5 meters in all study sites. The occurrence of the 1998 and 2010 coral bleaching events, the tsunami in 2004 and outbreaks of *A. planci* in 2015 is shown with arrows. Data for 2016 were collected during the present study, while historical data on study sites from 1997 to 2013 were extrapolated from^6^,^29^,^31^,^41^,^42^,^46^,^57^,^70^,^74^,^75^,^76^,^77^,^78^,^79^,^80^.

Mean coral cover declined from 40.08% (±12 95% CL) in 1997 to 1.69% (±3.59 95% CL) in 1998, due to the mass-bleaching. Since that time, coral cover increased fairly consistently until 2012 (Fig. 1). The average annual rate of change, across all sites was 93.5% ± 3.08, and did not vary significantly between lagoon versus oceanic island, nor with depth. Since 2012, there have been some marked declines in coral cover at some sites (Fesdu, Kuda Kandu), but not at others (Rasfari) due the ongoing outbreak of *A. planci* (Table S1). Overall, there has been a sustained increase in coral cover at sites surveyed in the Maldives since the 1998 mass bleaching (Fig. 1, Table S1), though recovery has been compromised in very recent years by other acute disturbances (Fig. 1).

Coral cover profiles varied among sites, with some sites never returning to pre-bleaching state and some showing lower cover than others (Table S1). Rasfari showed a significant decline after 1998, but then coral cover remained stable from 2005 to 2016 (Table S1). Similarly, in Udafushi coral cover declined significantly from 1997 (annual rate of change of 96.6% ± 36.8) and never recovered to pre-bleaching level, but instead remained quite low (Table S1). However, in most sites recovery trends reflected the patterns of disturbance over time (Table S1). Fesdu and Bandos for instance, showed two major declines corresponding with the 1998 bleaching event and the *A. planci* outbreak started in 2015 (annual rate of change from 2012 to 2016 of 82% ± 33.81 and 59.9% ± 4.2 respectively) (Table S1). Among all sites, Fesdu showed the fastest recovery following the 1998 bleaching event with an annual geometric rate of change from 2000 to 2002 of −164.51% ± 1.48 (Table S1).

**Figure 2:**
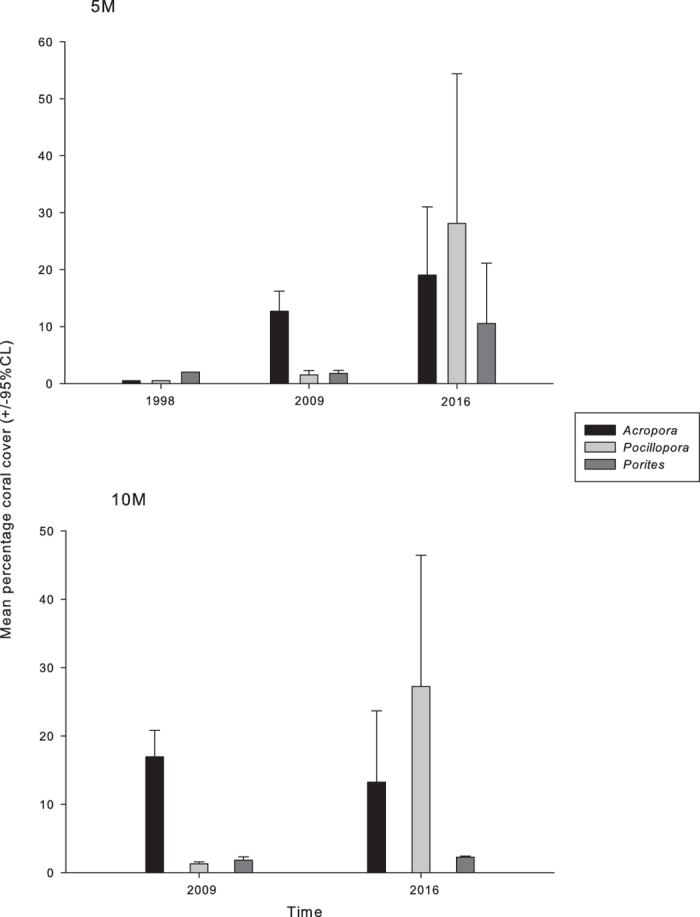
Temporal variation in mean percentage live coral cover (±95% CL) of major coral genera *Acropora, Pocillopora* and *Porites* at the study sites at 5 m and 10 m depth in 1998, 2009 and 2016.

Aside from causing marked changes in total live cover, acute disturbances occurring in Maldives from 1997 to 2016 changed the relative abundance of key genera through time (Fig. 2). In 1998, live cover of *Acropora* and *Pocillopora* was nearly 0%, while massive *Porites* was <2%. In 2009 live coral cover of branching and tabular *Acropora* increased to 12.6% ± 3.53 and was significantly higher than *Pocillopora* and *Porites* (1.51% ± 0.75 and 1.8% ± 0.51 respectively) (Fig. 2). *Acropora* showed an upward trend until 2016 however during the outbreak of *A. planci* live cover was lower that the other taxa and started to decline at 10 m (Fig. 2).

### Coral size-frequency distributions

**Figure 3:**
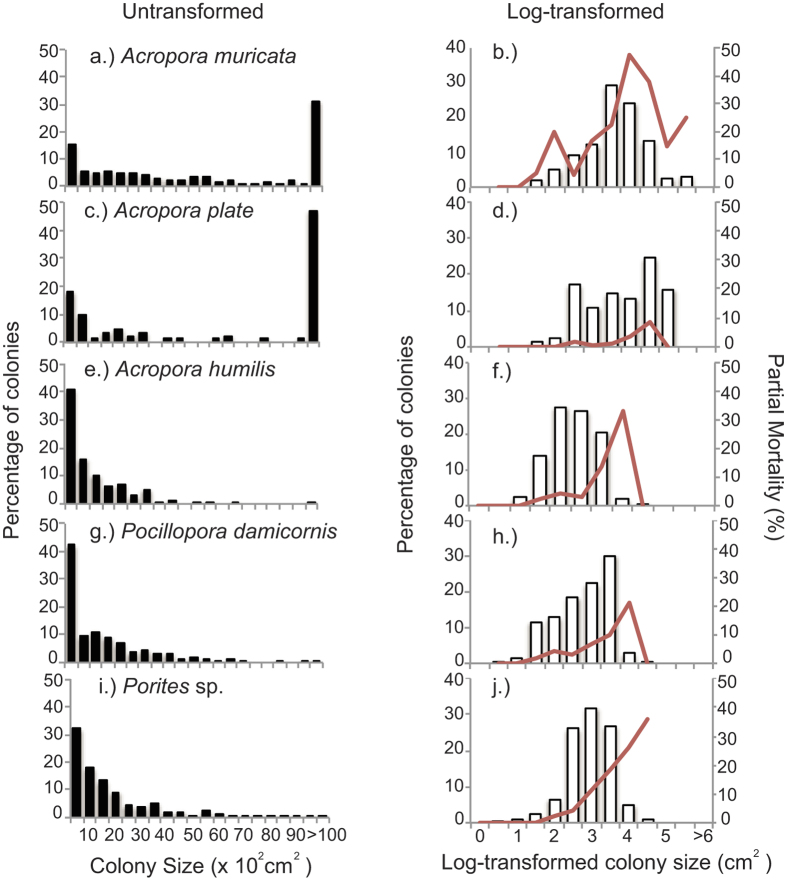
Log-transformed and untransformed size-frequency distributions of *Acropora muricata*, tabular *Acropora*, *Acropora humilis*, *Pocillopora* spp and *Porites* spp. The dark line indicates average partial mortality (%) for each size class.

A total of 1966 colonies were surveyed in 2016, across 42 transects at 7 sites. The structure of coral populations varied significantly among taxa (ANOVA, F_7,1922_ = 44.56, p = <0.001). Based on Tukey’s post hoc test, *A. muricata* surface area was significantly larger than all other species reaching 3846.45 cm (±17.66 SE), while *Porites* spp had the smallest colony size 0.04 cm (±4.93 SE). Within taxa, size-frequency distributions did not vary between depths or between inner versus outer islands (KS test, P > 0.01). When using the untransformed data, there was a prevalence of smaller colonies (Fig. 3) in all coral taxa except for *A. muricata*, resulting in positively skewed size-frequency distributions (Fig. 3). *A. muricata* had a larger percentage (62%) of colonies in the largest class size (>10.000 cm^2^) compared to the other species (Fig. 3). By log-transforming the data, the size-frequency distributions became more normally distributed compared to the untransformed data (Fig. 3). Transformed size-frequency distributions for all coral taxa were negatively skewed, with a preponderance of colonies in the largest size-classes (Fig. 3, Table 1). Size-frequency distributions were also leptokurtic, peaked and highly centralized around the mean, indicative of slower population growth (Fig. 3, Table 1).

Tabular *Acropora* had the greatest mean colony size (4.57 cm^2^), followed by *A. muricata* (4.07 cm^2^), while *Porites* spp (3.35 cm^2^) had similar to that of *Pocillopora* (3.34 cm^2^) (Table 1). The CV ranged from 19.9 for *Porites* spp, to 27.1 for *Pocillopora* (Table 1). The total range of skewness (g_1_) was −0.01 (for *A. humilis*) to −0.49 (for *Pocillopora*). Kurtosis (g_2_) was highly positive for all the species ranging from 2.34 in to 3.15 in *A. muricata*.

In *Porites* spp, tabular *Acropora* and *A. muricata* percentage of partial mortality increased as the colonies became larger (Fig. 3), while in *Pocillopora* spp and *A. humilis* percentage of partial mortality increased with increasing size but then decreased in the largest size classes (Fig. 3). Mean percentage of dead tissue was highest in *A. muricata* and *Porites* (27.1% ± 2.3 and 11.3% ± 0.6 respectively) while it was lowest in tabular *Acropora* (3.1% ± 1.3) (Fig. 3).

Mean colony surface area differed significantly among the study sites for virtually all coral taxa (all except *A. muricata*) (Table 2; Tukey test > 0.005). Spatial variation in colony surface was not related to depth and was generally similar between oceanic versus lagoonal reefs (Table 2). However, colonies were slightly larger colonies on oceanic reefs for both *Porites* and *Pocillopora* (Tukey test < 0.05).

## Discussion

This study shows that coral assemblages in the Maldives recovered (albeit relatively slowly) in the aftermath of the 1998 mass coral bleaching event, whereby coral cover increased from 1.69% (±3.59) to 37.4% (±1.03) by 2009 (average annual rate of change in coral cover was 93.5% ± 3.08). Prior to the 1998 mass-bleaching, coral assemblages in the Maldives were mostly dominated by *Acropora*^40^ while after the 1998 mass bleaching the dominant corals were bleaching-tolerant massive and sub-massive genera such as *Porites* and *Pavona*^41^,^42^,^43^. The1998-bleaching event essentially extirpated the temperature sensitive genera, such as *Acropora*, *Pocillopora* and *Montipora*^41^,^44^. Given the spatial extent of coral loss, and especially the localized depletion of fast growing coral genera (e.g., *Acropora*), coral recovery in the Maldives has been relatively slow^43^. On Kenyan reefs *Pocillopora* and *Acropora* started to recruit in 2001^45^, whereas recruits of *Acropora* and *Pocillopora* where not apparent in the Maldives until 2009–2014^31^, and in 2001 *Pavona* was the most dominant recruit^46^. Rapid recovery of degraded reefs is largely dependent on the growth of remnant corals^5^,^30^ whereas recruitment and subsequent growth of new colonies can greatly extend recovery times. Initially slow rates of coral recovery in the aftermath of the 1998 bleaching in the Maldives reflect the widespread loss of fast growing corals, which is likely to occur more often and across a wide range of reef locations with ongoing increases in global warming and increasing incidence of mass-bleaching^30^,^41^,^43^,^47^,^48^.

Research on coral reef disturbances, and corresponding changes in the structure of coral populations and communities, almost invariably focuses on large-scale, unpredictable and acute disturbances^27^, essentially overlooking more chronic disturbances that can have important impacts on population and community dynamics^1^,^11^,^22^,^24^. Similarly, there is very limited effort to quantify chronic disturbances in Maldives (e.g., fishing pressure, sedimentation, and/or eutrophication), let alone assessing potential impacts (e.g., rates of background injury and mortality, or the extent to which coral growth is suppressed) on corals. It also appears that loss of corals is largely explained by the occurrence of acute disturbances, such as mass- bleaching, tsunamis, and outbreaks of *A. planci*, all of which have contributed to significant coral depletion in other locations in the Indian Ocean and Pacific^27^. Importantly, Maldives are experiencing rapidly increasing human pressures due to coastal development and ongoing increases in tourism^37^, such that there is a definite need to implement a systematic and sustained monitoring program, both to document specific and cumulative effects of increasing disturbances and identify effective management solutions.

Coral cover in the Maldives has generally increased in the period since the 1998 mass bleaching, but still very little is known about the longer term (multi-decadal) trajectories in coral assemblages, nor the historical structure of coral assemblages and level of coral cover, which provide an important reference for assessing coral recovery and reef resilience. Studies that documented extensive coral loss caused by the 1998 bleaching in the Maldives predicted that it would take 10–15 years with relatively few major disturbances for coral cover to return to 1997 levels^49^,^50^,^51^, which is supported by empirical data presented in the current study. However, there was significant spatial and temporal variation in rates of recovery, partly due to the occurrence of other disturbances. In addition to the 1998 mass bleaching, reefs in the central Maldives archipelago were affected by a minor tsunami, a mild bleaching event in 2010 and a significant and emerging outbreak of *A. planci* on Maldivian reefs^29^,^31^. There were not however, any major storms during this period^29^,^52^, which have contributed significantly to coral loss in other locations. Outbreaks of *A. planci* are one of the principal causes of coral loss in the Indo-Pacific^27^,^53^, often killing up to 90% of scleractinian corals^54^. Outbreaks of *A. planci* were previously reported in the Maldives in the 1990’s^55^, however densities of crown-of-thorns starfish recorded in recent years (2014–15) are the highest ever reported in the Maldives^56^.

Coral reefs in the Maldives are highly vulnerable to climate change (according to NOAA the threshold for coral bleaching is just below 31°) and are exposed to high levels of human stress^27^. Recovery of coral assemblages following the 1998 mass bleaching has been variable in terms of the time taken for the re-establishment of both pre-disturbance coral cover and composition^29^,^31^. In 1997, mean coral cover in the Maldives was 40.08% ± 12.07^29^,^31^,^41^,^57^, and only returned to these levels in 2012 (42.1% ± 1.26) before the effects of *A. planci* outbreaks started to become apparent. The rate of recovery in the aftermath of the mass-bleaching in the Maldives is slow compared to rates of recovery documented in the Chagos archipelago^58^ and other remote Indian Ocean locations^30^ with similar oceanographic conditions. Importantly, pre-bleaching coral composition has still not been restored at many locations in the Maldives^31^. However, general (albeit gradual) increases in the coral cover recorded in the Maldives since the 1998 mass-bleaching are a stark contrast to sustained declines in live coral cover recorded at some other well-studied reef locations^27^,^28^. In the Caribbean, as well as on Australia’s Great Barrier Reef (GBR), cumulative effects of major disturbances combined with chronic disturbances (e.g., overfishing and declines in water quality) have resulted in systematic and sustained declines in coral cover over the last 2–3 decades^27^,^28^,^59^,^60^. On the GBR, for example, reef-wide coral cover has declined by 50% over the last 27 years^27^, and experienced a further significant drop in 2016, due to the severe mass bleaching in 2016^39^. The limited capacity for coral recovery in these regions is attributed to the short return times of major disturbances^27^,^59^, as well as the effects of chronic, usually anthropogenic, impacts^28^ such as overfishing, sedimentation, and eutrophication, which constrain growth and/or reproduction of corals. Ongoing resilience of coral assemblages in the Maldives, necessary for recovery between successive major disturbances, is therefore conditional upon effective management of anthropogenic activities to minimize chronic disturbances.

Spatial variation (among sites) in recovery of coral assemblages in the Maldives partly reflects inherent variation in coral composition, and specifically the functional composition of coral assemblages. The sites where coral cover increased most rapidly following the 1998 bleaching event were generally dominated by small and fast growing corals (47.6% ± 0.07 which was the 66% of the total coral cover), whereas recovery was very slow at sites dominated by large colonies of slow-growing corals, such as massive *Porites* (17% ± 0.01 which was the 56% of the total coral cover). Such differences in coral composition may have been structured by differences in the disturbance regime and history among sites, but also reflect spatial variation in environmental conditions that have selective effects on coral composition. Notably, the site in South Male atoll is subject to high levels of sedimentation, partly caused by extensive sand mining at nearby locations (Rilwan and Najeeb personal observations), which tends to have disproportionate negative affects on branching corals^37^,^61^. Spatial variation in management regimes, and corresponding differences in the extent of anthropogenic activities (mainly, fishing), may also influence the resilience of local coral assemblages^37^,^62^. Fesdu, Velidhu and Bandos are closed to fishing, but close to major resorts, whereas all the other sites are open to fishing, but do not have any resident human populations on the associated islands. Both coral cover and rates of coral recovery were higher at the tourist islands (Fesdu, Velidhu and Bandos), compared to uninhabited islands, suggesting that inhabitation of islands in itself does not necessarily constrain coral reef resilience. Rather, anthropogenic activities, such as fishing and eutrophication, need to be carefully managed.

Aside from temporal changes in coral cover, the present study also explored the size-frequency distribution of five major dominant coral taxa, providing significant insights into potential effects of recent disturbances and the likely future for these dominant and ecologically important coral populations. The size-frequency distributions of all coral species were dominated by larger size classes with over-centralized, peaked distributions (negatively skewed with positive kurtosis) indicating that the smaller size classes were generally under-represented. Observed size-frequency distributions departed from normal expectations^63^, especially for the faster growing corals (*Acropora*, and *Pocillopora* spp), and suggest that either there has been ongoing suppression of reproduction and recruitment, or that recent disturbances have caused disproportionate mortality among smaller size classes^64^,^65^. Recruitment rates of *Acropora* and *Pocillopora* were high in 2014^31^, which likely reflects recent increases in the local abundance of large colonies that have a disproportionate contribution to reproduction^36^. However, elevated rates of mortality among smaller size classes are concerning because it constrains population turnover, and makes local coral populations extremely susceptible to the elevated mortality of the larger, longer-lived colonies, especially given recent mass-bleaching in the Maldives^39^.

In this study, partial mortality increased with colony size in *Porites* spp, tabular *Acropora* and *A. muricata.* Like fragmentation, partial mortality is critical in determining the size of coral colonies, as the loss of living tissue can cause colonies to regress in size^66^. Exposure to the specific agents of partial mortality tends to increase with colony size, but the likelihood that a given disturbance or injury will cause whole-colony mortality is lower in larger colonies^33^. Different incidence of partial mortality among size classes observed here, suggests size-specific susceptibility to agents of coral mortality such as sedimentation, predation, fragmentation and competitive interactions^34^. Higher rates of tissue loss with increasing size may also be due to accumulation of old injuries. Regeneration of injuries is often incomplete and larger and therefore older colonies may have more time to accumulate multiple old lesions^67^. The energetic costs of regeneration likely vary with different size classes. An injury on a small individual affects a greater area than on larger colonies and is likely to have a higher energetic cost in terms of regeneration due to limited resources available within a colony^67^.

While there was significant spatial variation in mean colony size of all coral taxa (except *A. muricata*), this study did not detect any clear and consistent difference between depths and between exposed and less exposed locations. Only *Porites* and *Pocillopora* showed slightly larger colonies in the oceanic reefs compared to the lagoonal ones. These findings may suggest that other factors including disturbance regime and life history processes further modify the structure of coral populations. Differences in recruitment, growth, partial and total mortality rates may also cause spatial variation in the size structure as they can vary with small spatial scale^68^.

This study documented long-term changes in coral communities and showed how disturbance regime structured size frequency distribution in the focal species. We showed that Maldivian systems are slowly capable of recovery following multiple disturbances, but this does not guarantee that theses reefs will be resilient to further disturbances. Recent outbreaks of *A. planci* (since 2012) have certainly contributed to coral depletion, and these reefs were again subject to extensive mass bleaching in 2016^39^, which will likely lead to highly protracted recovery times. With increasing frequency of acute disturbances and escalating effects of climate change and human pressure, it is important to increase understanding on ecosystem recovery capacity, and changes in communities^69^. Degradation and loss of coral reef ecosystems has serious ramifications for structure and dynamics of reef communities, such that knowledge of long term changes in coral composition and life-history dynamics is fundamental to effective ecosystem management. Changes in habitat quality and quantity need to be evaluated and long-term monitoring is the key to understand the future evolution of coral reef ecosystems.

## Materials and Methods

### Study site

The Maldives comprise 16 complex atolls with ca.1120 islands arranged along the Chagos-Maldives-Laccadive ridge in the central Indian Ocean from about 7°07′ N to 0°40′ S. This study surveyed 7 islands in three atolls in the central Maldives archipelago. A total of three islands with exposed ocean-facing reefs on the atoll rim and four islands inside the sheltered atoll lagoon were considered to test for differences in contemporary habitat structure based on reef typology. In February-March 2016, the cover and composition of corals, as well as size structure for six distinct coral taxa (*Acropora hyacinthus, Acropora cytherea, Acropora muricata, Acropora humilis, Pocillopora spp,* and massive *Porites*) were measured at two depths (5 m and 10 m) at each of 7 islands (Table 3). The focal species were selected based on their reported abundance across all study sites^70^ but also because of their contrasting morphologies and life-history strategies^69^. While it would have been ideal to distinguish individual species, and thereby capture species-specific differences in their ecology, it was necessary to pool data for *Pocillopora* and *Porites* spp. due to difficulties in consistently differentiating species in the field^71^,^72^,^73^.

To measure size structure of coral species, three replicate 10 × 2 m belt transects were laid parallel to the reef edge, on both the reef crest (5 meters) and slope (10 meters). For every colony sampled, the maximum diameter was recorded. Partial mortality (percentage of tissue loss) was also visually estimated *in situ* to the nearest 5% for each surveyed colony and causes of injuries were also recorded, where possible following Pisapia *et al.*^11^. Estimates of total coral cover and composition (relative abundance of all genera) were derived using line transect methods along the fiberglass tape used to delineate each belt transect.

### Long-term changes in coral cover

Recent changes in coral cover and composition were explored by compiling data from 14 distinct studies^6^,^29^,^31^,^41^,^42^,^46^,^57^,^70^,^74^,^75^,^76^,^77^,^78^,^79^,^80^, which provide historical estimates of total coral cover, largely based on single surveys at individual locations between 1993 and 2014 70,75,79. There has however, been routine monitoring at five of the sites considered in this study, using mostly consistent methods since 1998^29^,^31^,^46^,^57^,^79^, prompted by the desire to document recovery of coral assemblages following the 1998 mass-bleaching^29^. Initial surveys were conducted in 15 sites across 6 atolls at 5 m almost every year until 2005 using three replicate 50-m line intercept transects. Since 2009 data were collected in the same sites at both 5 m and 10 m depths, but using four replicate 20-m point intercept transects^79^.

To explore long-term changes in coral community structure at the study sites, we compiled quantitative data from the literature combining those with the data recorded during this study. Past data at the study sites were only available for branching and tabular *Acropora*, *Pocillopora* spp and massive *Porites* spp.

### Data analyses

The temporal occurrence and severity of major disturbances such as coral bleaching, tsunami, management regime and outbreaks of crown-of-thorns *A. planci* (COTS) was assessed with published literature. To quantify total coral cover trends, annual geometric rate of change in coral cover for each year was calculated following Côté *et al.*^60^. Geometric rates of change in live coral cover were calculated based on the relative change in coral cover between respective samples, and it was taken into account that the temporal changes in coral cover were nonlinear following Côté *et al.*^60^ (e.g., considering change from year to year in exponentially declining or increasing coral cover). The confidence intervals were calculated using the R package ‘propagate’.

Differences in coral cover trend from 1997 to 2016 between outer and inner islands, between 5 m and 10 m and between sites were analyzed using a Generalized Least Squares model. Coral cover was the dependent variable while island position, water depth and time were the predictors. Year, exposure, and depth were treated as fixed factors, while site as random. Models were compared by maximum likelihood and the model with the lowest Akaike information criterion (AICc) was chosen as the best representation of the variation in the data.

To better understand consequences of recent disturbances on the populations structure of dominant coral taxa, we examined size-frequency distributions. The maximum diameter (cm) was used to approximate the 2-dimensional projected surface area of each coral colony, following Linares *et al.*^13^. Colony surface area (cm^2^) was converted to living area by subtracting the percentage of mortality for each colony. Colony surface-area data were log_10_ transformed to normalize size- frequency distributions and increase resolution among smaller size classes, following Bak and Meesters^34^.

Variation in the population structure of dominant coral taxa between depths (5 m versus 10 m) and between reefs (outer islands vs inner islands) was investigated using 2- sample Kolmogorov–Smirnov (KS) tests for each species separately. Variation in colony sizes, coefficient of variation, (CV), skewness (g_1_) and kurtosis (g_2_) were calculated for each species. The geometric mean provides relative measures of colony size providing information relevant to other key demographic process, such as reproductive output^34^. The CV is the standard deviation as percentage of the mean and describes the variation in the data set and allows for comparisons irrespective of the mean. Skewness describes the relative abundance of colonies that are smaller or larger than the geometric mean. If the skewness is negative, the population is skewed to the left, with a relatively larger proportion of colonies in the larger size classes than in the smaller size classes^34^. If the skewness is positive the population is skewed to the right, containing a larger number of individuals in the smaller size classes. Kurtosis describes the concentration of data around the central mode of a distribution among populations indicating whether the data is peaked or flat relative to the normal distribution. If kurtosis is negative, the distribution is platikurtic with a wide peak around the mean. Conversely, a positive kurtosis indicates the distribution is leptokurtic, which is peaked and highly centralized around the mean.

Differences in surface area of living tissue (SA) among sites were simultaneously tested for each coral taxa using a Three-ways ANOVA with surface area as dependent variable and sites, reef typology (lagoon vs oceanic) and depth (5 m and 10 m) as independent. In all the analyses *A. hyacinthus* and *A. cytherea* were pooled because sample sizes were small (less than 100 individuals for each species) and demographics expected to be very similar for these two “tabular” corals. Difference in surface area of living tissue (SA) among species were tested using a One-way ANOVA and a Tukey’s post hoc test was then utilized to determine specific differences among species. All the analyses were run using R vs 3.3.1.

## Additional Information

**How to cite this article**: Pisapia, C. *et al.* Coral recovery in the central Maldives archipelago since the last major mass-bleaching, in 1998. *Sci. Rep.* **6**, 34720; doi: 10.1038/srep34720 (2016).

## Supplementary Material

Supplementary Information

## Figures and Tables

**Table 1:** Statistical summary of the size-frequency distributions (using log-transformed colony size) for 5 coral taxa.

Species	n	Mean colony size (cm^2^)	CV	Kurtosis	Skewness
*Acropora muricata*	221	4.07	22.9	3.15	−0.21
*Acropora humilis*	174	3.22	20.4	2.57	−0.01
Tabular *Acropora*	83	4.57	25.9	2.38	−0.1
*Pocillopora spp*	525	3.34	27.1	2.34	−0.49
*Porites*	968	3.35	19.9	3.59	−0.41

The sample size (n), density, log-transformed mean colony size, coefficient of variation (CV), skewness (g_1_), kurtosis (g_2_) are specified.

**Table 2:** Statistical summary of three-ways Anova for 5 coral taxa using mean colony size, sample sites, site exposure and depth as variables.

	df	SS	F	p
*Acropora muricata*				
Site	3	19574	2.01	0.11
Exposure				
Depth	1	18079	0.05	0.81
Error	194	63235		
*Acropora humilis*				
Site	5	37323	10.7	***
Exposure	3	7878	3.09	0.03
Depth	1	652	0.93	0.33
Error	155	10781		
*Acropora plate*				
Site	3	30784	12.1	***
Exposure				
Depth	1	31661	0.37	0.5
Error	78	65956		
*Pocillopora spp*				
Site	6	49858	17.3	***
Exposure	2	9307	8.39	***
Depth	1	637	1.32	0.24
Error	517			
*Porites*				
Site	6	48179	9.8	***
Exposure	3	34639	13.86	***
Depth	1	3500	4.27	0.24
Error	959	7852		

**Table 3:** Study sites and atolls with indicated the presence or absence of human settlement (uninhabited or resort), the two types of locations (lagoon or oceanic reefs), latitude, and longitude.

Atoll	Type	Site	Position	Latitude	Longitude
North Male	Uninhabited	Udhafushi	Lagoon	4 18.47′N	73 30.14′E
North Male	Resort	Bandos	Lagoon	4 16.26′N	73 29.29′E
Ari Atoll	Resort	Velidhu	Lagoon	4 11.34′N	72 49.10′E
Ari Atoll	Resort	Fesdu	Lagoon	4 0.31′N	72 48.35′E
North Male	Uninhabited	Rasfari	Oceanic	4 36.19′N	73 35.90′E
South Male	Uninhabited	Emboodhu	Oceanic	4 7.77′N	73 28.19′E
North Male	Uninhabited	KudaKandu	Oceanic	4 36.19′N	73 35.90′E
