# Supplementary material for: Coral recovery in the central Maldives archipelago since the last major mass-bleaching, in 1998
Source: Sci Rep. 2016 Oct 3;6:34720. doi: 10.1038/srep34720 (PMC5046149; doi:10.1038/srep34720)
Supplement: Supplementary Information [file srep34720-s1.doc]

**Coral recovery in the central Maldives archipelago since the last major mass-bleaching, in 1998**

Pisapia C^1^*, Burn D^2^, Rilwan Y^3^, Najeeb A^3^, Anderson K^1^, Pratchett MS^1^

1 ARC Centre of Excellence for Coral Reef Studies, James Cook University, Townsville QLD 4811, Australia

2 Gili Lankanfushi Island, North Male Atoll, Republic of Maldives

3 Marine Research Centre, Ministry of Fisheries Agriculture and Marine Resources, Moonlight Hingun, 20025, Male’ Republic of Maldives

*corresponding author: chiara.pisapia@my.jcu.edu.au

**Keywords**: Long-term changes; Coral cover; Coral size structure; Recurrent stressors; Coral reefs; Indian Ocean

**Table S1.** Supplementary table showing annual rates of change (%) (±95% CI) in live coral from 1997 to 2016 for every site at both 5m and 10m.

| **Atoll** | **Site** | **Exposure** | **Depth** | **Year** | **Geom rate of change** | **2.5%** | **97.5%** |
| --- | --- | --- | --- | --- | --- | --- | --- |
| North Ari | Fesdu | Inner | 5 | 1998 |  |  |  |
| North Ari | Fesdu | Inner | 5 | 1999 | -12.36 | -10 | 17.7 |
| North Ari | Fesdu | Inner | 5 | 2000 | -164.51 | -0.48 | 1.46 |
| North Ari | Fesdu | Inner | 5 | 2002 | -106.85 | -0.75 | 1.08 |
| North Ari | Fesdu | Inner | 5 | 2004 | -23.27 | 0.33 | 1.28 |
| North Ari | Fesdu | Inner | 5 | 2005 | -15.91 | 0.42 | 1.42 |
| North Ari | Fesdu | Inner | 5 | 2009 | -102.64 | 0.22 | 0.74 |
| North Ari | Fesdu | Inner | 5 | 2012 | 38.69 | 1 | 1.03 |
| North Ari | Fesdu | Inner | 5 | 2016 | 82.05 | 1.8 | 2.6 |
| North Ari | Fesdu | Inner | 10 | 2009 |  |  |  |
| North Ari | Fesdu | Inner | 10 | 2012 | 36.11 | 0.96 | 1.13 |
| North Ari | Fesdu | Inner | 10 | 2016 | 80.52 | 1.38 | 2.1 |
| North Ari | Velidhoo | Inner | 5 | 1998 |  |  |  |
| North Ari | Velidhoo | Inner | 5 | 1999 | 20 | -20.1 | 35.5 |
| North Ari | Velidhoo | Inner | 5 | 2000 | -314.58 | -1 | 1.71 |
| North Ari | Velidhoo | Inner | 5 | 2002 | -244.72 | -2.49 | 5.83 |
| North Ari | Velidhoo | Inner | 5 | 2004 | -0.29 | -3.58 | 5.85 |
| North Ari | Velidhoo | Inner | 5 | 2005 | -137.79 | 0.1 | 0.73 |
| North Ari | Velidhoo | Inner | 5 | 2009 | -151.72 | 0.09 | 0.12 |
| North Ari | Velidhoo | Inner | 5 | 2012 | 56.09 | 1.12 | 1.3 |
| North Ari | Velidhu | Inner | 5 | 2016 | -81.2 | 0.6 | 0.7 |
| North Ari | Velidhoo | Inner | 10 | 2009 |  |  |  |
| North Ari | Velidhoo | Inner | 10 | 2012 | -46 | 0.75 | 1.15 |
| North Ari | Velidhoo | Inner | 10 | 2016 | -93.23 | 0.44 | 0.73 |
| North Male | Bandos | Inner | 5 | 1997 |  |  |  |
| North Male | Bandos | Inner | 5 | 1998 | 94.83 | -2.21 | 53.05 |
| North Male | Bandos | Inner | 5 | 1999 | -292.47 | -1.27 | 3.26 |
| North Male | Bandos | Inner | 5 | 2000 | 20.26 | -5.54 | 11.4 |
| North Male | Bandos | Inner | 5 | 2002 | -14.87 | -3 | 7.31 |
| North Male | Bandos | Inner | 5 | 2009 | -125.66 | -0.23 | 0.56 |
| North Male | Bandos | Inner | 5 | 2011 | -13.59 | 0.84 | 0.91 |
| North Male | Bandos | Inner | 5 | 2012 | 27.99 | 1.3 | 1.47 |
| North Male | Bandos | Inner | 5 | 2016 | 60 | 1.01 | 1.29 |
| North Male | Bandos | Inner | 10 | 2011 |  |  |  |
| North Male | Bandos | Inner | 10 | 2012 | -0.04 | 1.15 | 1.36 |
| North Male | Bandos | Inner | 10 | 2016 | -0.19 | 0.64 | 0.75 |
| North Male | KudaKandu | Outer | 10 | 1993 |  |  |  |
| North Male | KudaKandu | Outer | 10 | 2016 | 96.73 | -0.9 | 6.21 |
| North Male | Rasfari | Outer | 5 | 1997 |  |  |  |
| North Male | Rasfari | Outer | 5 | 2016 | 96.43 | -0.44 | 6.11 |
| North Male | Rasfari | Outer | 10 | 1993 |  |  |  |
| North Male | Rasfari | Outer | 10 | 2005 | 95.64 |  |  |
| North Male | Rasfari | Outer | 10 | 2016 | -82.91 | 0.85 | 0.93 |
| North Male | Udhafushi | Inner | 5 | 1997 |  |  |  |
| North Male | Udhafushi | Inner | 5 | 1998 | 96.65 | -696.8 | 1537.12 |
| North Male | Udhafushi | Inner | 5 | 1999 | -12.02 | -9.07 | 13.84 |
| North Male | Udhafushi | Inner | 5 | 2000 | -46.35 | -1.95 | 4.57 |
| North Male | Udhafushi | Inner | 5 | 2002 | -34.17 | -1.01 | 3.01 |
| North Male | Udhafushi | Inner | 5 | 2009 | -129.74 | -0.02 | 0.3 |
| North Male | Udhafushi | Inner | 5 | 2011 | 69.67 | 1.49 | 2.45 |
| North Male | Udafushi | Inner | 5 | 2016 | 85.82 | 1.35 | 2.45 |
| North Male | Udhafushi | Inner | 10 | 2009 |  |  |  |
| North Male | Udhafushi | Inner | 10 | 2011 | 39.43 | -0.54 | 3.88 |
| North Male | Udafushi | Inner | 10 | 2016 | -76.26 | 0.6 | 0.99 |
| South Male | Emboodhoo | Outer | 5 | 1998 |  |  |  |
| South Male | Emboodhoo | Outer | 5 | 1999 | -33.99 | -10.7 | 23.7 |
| South Male | Emboodhoo | Outer | 5 | 2000 | 19.3 | 24.1 | 46.2 |
| South Male | Emboodhoo | Outer | 5 | 2002 | -3.12 | -6.79 | 11 |
| South Male | Emboodhoo | Outer | 5 | 2004 | -19.22 | -1.19 | 3.28 |
| South Male | Emboodhoo | Outer | 5 | 2009 | -130.23 | 0 | 0.42 |
| South Male | Emboodhoo | Outer | 5 | 2011 | -294.34 | 0.18 | 0.32 |
| South Male | Emboodhoo | Outer | 5 | 2012 | 22.25 | 1.15 | 1.42 |
| South Male | Emboodhoo | Outer | 5 | 2016 | -65.58 | 0.71 | 0.85 |
| South Male | Emboodhoo | Outer | 10 | 2009 |  |  |  |
| South Male | Emboodhoo | Outer | 10 | 2011 | -78.64 | 0.51 | 0.6 |
| South Male | Emboodhoo | Outer | 10 | 2012 | 20.19 | 1.11 | 1.4 |
| South Male | Emboodhoo | Outer | 10 | 2016 | 64.35 | 1.11 | 1.3 |
|  |  |  |  |  |  |  |  |
